# One Anastomosis Gastric Bypass versus Roux-en-Y Gastric Bypass: A Randomized Prospective Trial

**DOI:** 10.3390/medicina60020256

**Published:** 2024-02-01

**Authors:** Servet Karagul, Serdar Senol, Oktay Karakose, Kevser Uzunoglu, Cuneyt Kayaalp

**Affiliations:** 1 Department of General Surgery, Samsun Training and Research Hospital, 55090 Samsun, Turkey; serdarardaduru@gmail.com (S.S.); oktaykarakose@gmail.com (O.K.); dr.kevseruzunoglu@gmail.com (K.U.); 2 Private Clinic of Gastroenterological Surgery, 34363 Istanbul, Turkey; cuneytkayaalp@hotmail.com

**Keywords:** bariatric surgery, one anastomosis, Roux-en-Y gastric bypass

## Abstract

*Background and Objectives:* One anastomosis gastric bypass (OAGB) and Roux-en-Y gastric bypass (RYGB) surgeries are effective methods used in bariatric surgery. There are limited randomized studies comparing these procedures over more than 2 years. Here, we aimed to compare the 3-year results of two bariatric procedures. *Materials and Methods:* Patients included in this randomized prospective study were compared in OAGB and RYGB groups. A total of 55 patients, aged between 18 and 65, were eligible for the study. Thirteen patients who did not accept randomization were excluded. Patients were evaluated at 6, 12, 24, and 36 months postoperatively. *Results:* Three patients were excluded from the study due to loss of communication during the clinical follow-up and one due to death by amyotrophic lateral sclerosis, which started in the eighth month after surgery. The study was completed with a total of 38 patients (OAGB; *n* = 20, RYGB; *n* = 18). Patients in the two groups were similar in terms of age, gender, body mass index (BMI), and obesity-related comorbidities. At the end of 3-year follow-up, BMI in the OAGB and RYGB groups was 28.80 ± 4.53 kg/m^2^ and 29.17 ± 5.36 kg/m^2^, respectively (*p* = 0.822). Percentage total weight loss (TWL%) was similar. No significant differences were found between the groups regarding percentage excess weight loss (EWL%). Remission of comorbidities was similar. De novo refluxes developed in four OAGB patients; there were no occurrences of these in RYGB patients (*p* = 0.066). *Conclusions:* Both OAGB and RYGB are effective in the treatment of morbid obesity. The two procedures are similarly successful in terms of obesity-related comorbidities.

## 1. Introduction

In recent years, changes in lifestyle and diet type have had negative effects on the balance of energy in the human body. Physical inactivity, which is one of the most important of these changes, is a risk factor for weight gain. In addition, the food industry has contributed to the increase in obesity with the overconsumption of ultra-processed food [1,2,3,4,5]. In 2016, more than 1.9 billion adults aged 18 years and older were overweight, and over 650 million adults were obese. The worldwide prevalence of obesity nearly tripled between 1975 and 2016 [6]. It appears that these numbers will continue to rise. Regrettably, environmental conditions persistently impact people in an adverse manner. Obesity is now considered a global epidemic. However, we cannot say that there are effective solutions on the horizon to prevent people from becoming obese.

Obesity threatens human health not only with the presence of extra fat mass in the body but also with problems such as hypertension, diabetes, asthma, cardiovascular disease, sleep apnea, and joint disorders. In addition, obesity is known to be associated with many cancers, such as breast, pancreatic, stomach, endometrial, and colorectal cancers [7,8,9,10,11,12]. Obesity is also considered a disease that needs to be treated. Lifestyle changes are not yet as successful as bariatric surgery in the treatment of morbid obesity. Although success is achieved with lifestyle changes in the short term, in the long term, patients’ incompliance with diet or inability to maintain physical exercise leads to decreased weight loss in patients [13,14,15]. Medical treatments have been tried, and their studies are still ongoing. Semaglutide has provided promising results in the medical treatment of obesity and may open new horizons depending on long-term results [16,17]. Based on the data obtained, bariatric surgery seems to be the most successful method of morbid obesity treatment [18,19].

Different surgical treatment options have been applied from the past to the present. Roux-en-Y gastric bypass (RYGB) has been an option for many years. It has been accepted as a reliable bariatric surgery method with long-term results [20,21,22,23,24,25,26]. One anastomosis gastric bypass (OAGB), a simplified version of RYGB, was presented as a less complicated method [27]. Operative dissection is less practiced due to the absence of enteroenterostomy. In addition, complications such as bleeding of the second anastomosis line, internal herniation, and leakage from the second anastomosis are avoided with a single anastomosis [28,29]. Both surgeries are performed safely. However, there is no gold standard method among gastric bypass procedures in bariatric surgery yet. Although they have been used for decades, very few studies have compared OAGB and RYGB in a randomized fashion. To our knowledge, we present the third randomized study comparing OAGB and standard RYGB for up to 3 years in the literature [30,31]. Here, we aimed to compare the results of two procedures in terms of weight loss and obesity-related comorbidities.

## 2. Materials and Methods

**Figure 1:**
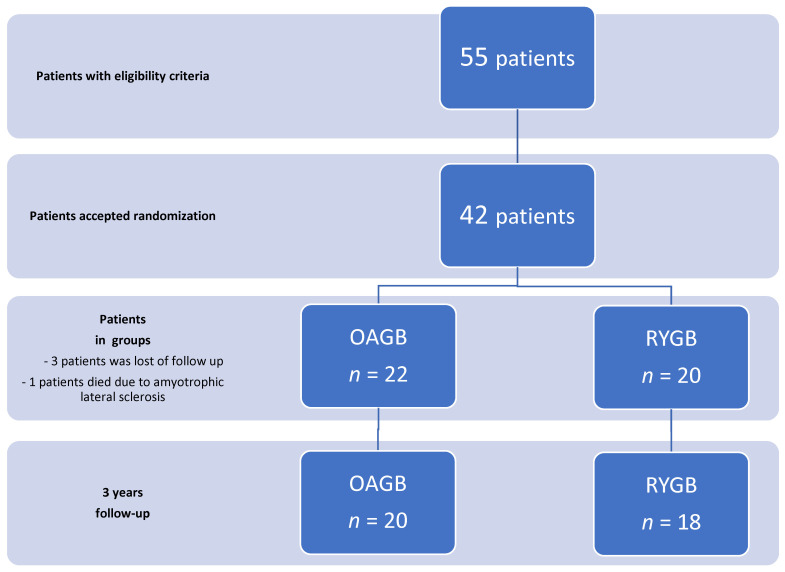
CONSORT diagram.

A randomized prospective single-center study was conducted in Samsun Training and Research Hospital, Turkey. This study was approved by the Ethics Committee of Samsun Training and Research Hospital (33646832-514.10) in accordance with the principles of the Declaration of Helsinki. All patients were evaluated by a multidisciplinary team consisting of an endocrinologist, psychiatrist, anesthesiologist, and general surgeon. In the study, two groups were compared, and one of the groups was the laparoscopic OAGB group and the other was the laparoscopic RYGB group. Patients with a BMI over 40 kg/m^2^ and patients with a BMI over 35 kg/m^2^ with obesity-related comorbidities were included. Morbid obese patients aged 18 to 65 years were eligible for the study. Substance abuse, hiatal hernia, gastroesophageal reflux, malabsorptive disease, pregnancy, severe heart failure, uncontrolled drug or alcohol dependency, Crohn’s disease, coagulopathy, and psychosis were the exclusion criteria. Written informed consent was obtained from all patients. Eligible candidates for bariatric surgery were informed about randomization. Those who accepted random surgery selection were randomized by a surgeon who did not perform the surgeries. Randomization was applied using the sealed envelope method. The operations were performed by 2 surgeons with vast experience in bariatric surgery. After the operation, postoperative diets were arranged by a dietitian. Patients were started on clear liquids on the first postoperative day. They were allowed to take liquid diet for 2 weeks. Then, they advanced to pureed diet for 4 weeks. At the end of these stages, the regular diet was started. Patients were discharged with a multivitamin supplement prescribed. Vitamin values were analyzed in clinic evaluations, and additional vitamin supplements were prescribed when necessary. Physical activity recommendations were made by an exercise specialist according to the physical characteristics of the patients. Patients were encouraged to start walking immediately after surgery. Four weeks later, aerobic exercises were started for at least 150 min per week to improve cardiovascular fitness. Two months after the operation, resistance exercises were added at least 2 days a week to prevent sarcopenia. Patients were called to the clinic 6, 12, 24, and 36 months after surgery. Information about patients who could not come to the clinic was recorded through phone calls. Patients who were lost of follow-up were excluded from the evaluation (Figure 1).

### 2.1. Surgical Techniques

**Figure 2:**
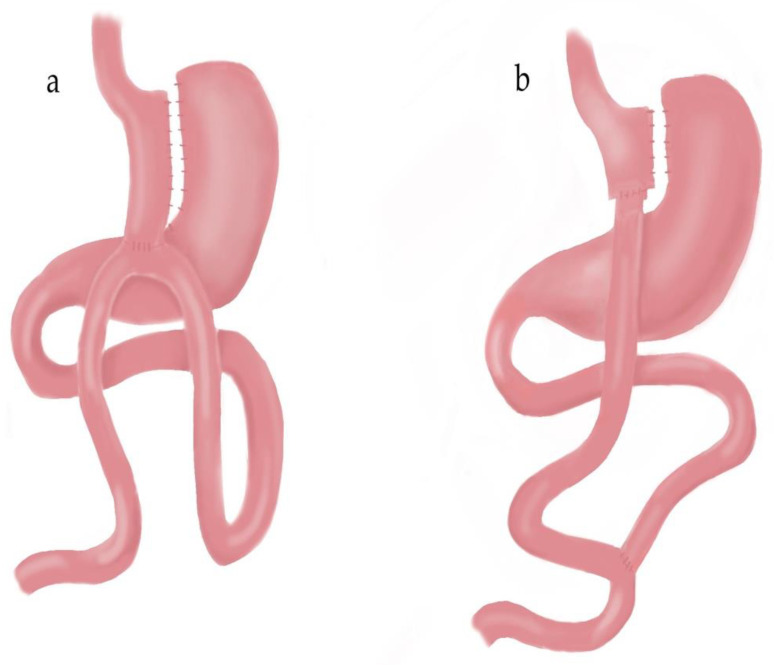
One anastomosis gastric bypass (**a**); Roux-en-Y gastric bypass (**b**).

In Roux-en-Y gastric bypass, a gastric pouch was created using an endoscopic stapler (EndoGIA, Medtronic, Minneapolis, MN, USA). An antecolic anastomosis was performed between the intestinal loop at 50 cm from the ligament of Treitz and the newly formed gastric pouch. The biliopancreatic limb was transected just proximal to the gastroenterostomy and anastomosed to 150 cm of the alimentary limb. Linear staplers were used in all steps of the operation. Resulting defects due to stapled anastomoses were closed by a double layer running suture technique. The mesenteric defect was closed using nonabsorbable sutures. Gastroenterostomy anastomosis was checked for leakage with methylene blue (Figure 2a).

In one anastomosis gastric bypass, dissection was carried out on the lesser curvature below the crow’s foot to enter into the lesser sac. A gastric pouch was created with the help of endoscopic staplers with the guidance of a gastric calibration tube. Anastomosis was created between jejunum at 200 cm from the ligament of Treitz and the new gastric pouch. Resulting defects due to stapled anastomosis were closed by a double layer running suture technique. Linear staplers were used in all steps of the operation (EndoGIA, Medtronic, Minneapolis, MN, USA). Gastroenterostomy anastomosis was checked for leakage with methylene blue (Figure 2b).

In both procedures, intestinal length measurements were made on the antimesenteric line without applying tension, and the intestinal sites where the anastomosis was performed were marked at the first measurement of the small bowel limbs. Total length of the small intestine was not measured.

### 2.2. Statistical Methods

Nominal and ordinal parameter descriptions were given by frequency analysis, whereas means and standard deviations were used for scale parameters. Fischer’s exact test was used for differences between categorical parameters. The Kolmogorov–Smirnov test was used for normality of the scale parameters. An independent samples *t*-test was used for normally distributed scale parameters, and the Mann–Whitney U test was used for non-normally distributed scale parameters. Wilcoxon’s signed rank test was used for nonparametric within-group comparisons, whereas a paired samples *t*-test was used for parametric within-group comparisons. All analyses were performed at SPSS 25.0 for windows at a 95% confidence interval with a 0.05 significance level. 

## 3. Results

A total of 55 patients, aged between 18 and 65, were eligible for the study. Thirteen patients who did not accept randomization were excluded. Forty-two patients were randomized to OAGB and RYGB groups. Three of the patients were excluded from the study due to loss of communication during the follow-up period, and one died due to amyotrophic lateral sclerosis, which started in the eighth month after surgery. The study was completed with a total of 38 patients randomized in OAGB (*n* = 20) and RYGB (*n* = 18) groups (Figure 1). Patients in the two groups were similar in terms of age, sex, and obesity-related comorbidities. The baseline body mass index (BMI) of the patients was 44.75 ± 6.10 kg/m^2^ in the OAGB group and 46.69 ± 6.62 kg/m^2^ in the RYGB group, and there was no difference (Table 1).

**Figure 3:**
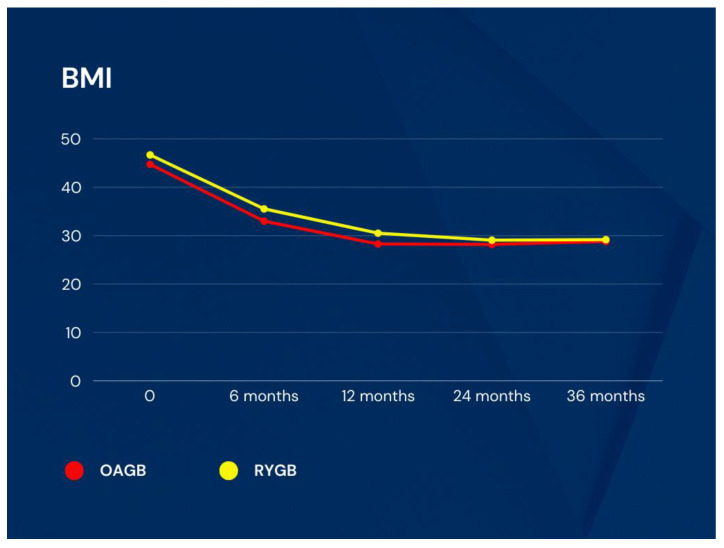
BMI changes in groups (kg/m^2^). BMI: body mass index, OAGB: one anastomosis gastric bypass, RYGB: Roux-en-Y gastric bypass.

In postoperative follow-up, there was no significant difference in BMI between the groups; BMI in the OAGB group vs. RYGB group was 33.01 ± 6.04 kg/m^2^ vs. 35.54 ± 5.62 kg/m^2^ (*p* = 0.190) at the 6th month, 28.28 ± 4.83 kg/m^2^ vs. 30.52 ± 4.50 kg/m^2^ (*p* = 0.149) at the 1st year, 28.20 ± 4.65 kg/m^2^ vs. 29.07 ± 5.06 kg/m^2^ (*p* = 0.584) at the 2nd year, and 28.80 ± 4.53 kg/m^2^ vs. 29.17 ± 5.36 kg/m^2^ (*p* = 0.822) at the 3rd year (Figure 3). 

**Figure 4:**
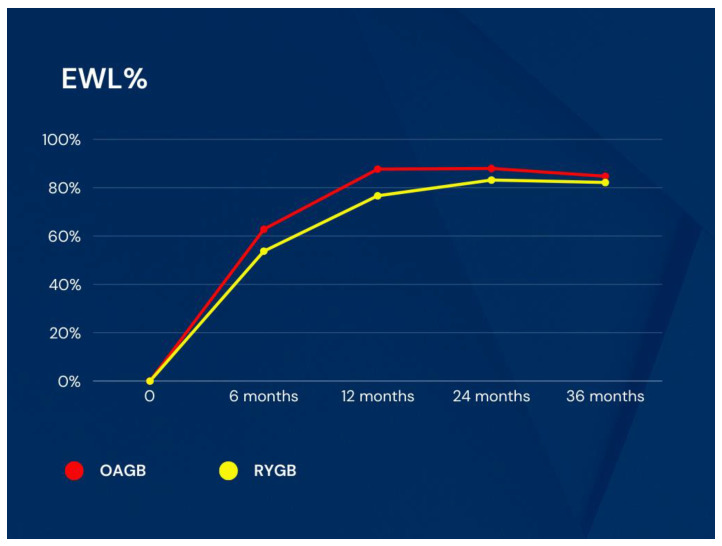
EWL% changes in groups. EWL: excess weight loss, OAGB: one anastomosis gastric bypass, RYGB: Roux-en-Y gastric bypass.

Percentage excess weight loss (EWL%) was 62.78 ± 24.44 vs. 53.73 ± 16.85 (*p* = 0.197) at the 6th month, 87.69 ± 20.53 vs. 76.66 ± 15.86 (*p* = 0.074) at the 1st year, 87.93 ± 19.20 vs. 83.17 ± 20.42 (*p* = 0.464) at the 2nd year, and 84.77 ± 18.90 vs. 82.15 ± 24.17 (*p* = 0.711) at the 3rd year, respectively (Figure 4).

* EWL% = [(initial weight – current weight)/(initial weight – ideal weight)] × 100

* The ideal weight was determined as the weight with a BMI of 25 kg/m^2^.

**Figure 5:**
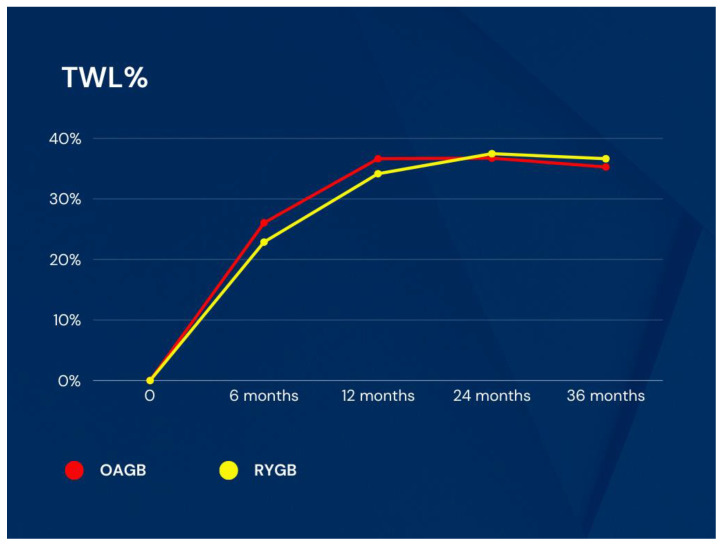
TWL% changes in groups. TWL: total weight loss, OAGB: one anastomosis gastric bypass, RYGB: Roux-en-Y gastric bypass.

Percentage total weight loss (TWL%) was 26.05 ± 9.10 vs. 22.87 ± 5.24 (*p* = 0.191) at the 6th month, 36.63 ± 6.84 vs. 34.16 ± 7.03 (*p* = 0.282) at the 1st year, 36.74 ± 6.20 vs. 37.47 ± 8.91 (*p* = 0.769) at the 2nd year, and 35.28 ± 5.35 vs. 36.64 ± 10.25 (*p* = 0.619) at the 3rd year, respectively (Figure 5).

* TWL% = [(initial weight − current weight)/(initial weight)] × 100

Type 2 diabetes mellitus (T2DM) remission was 92% in OAGB and 70% in RYGB, but no statistical difference was observed (*p* = 0.263). Medication for hypertension was discontinued in 66% of patients in both groups. Obesity-related comorbidity changes in patient groups were similar, and differences were statistically insignificant (*p* > 0.05). While four de novo refluxes developed in OAGB patients, there were none in RYGB patients (*p* = 0.066) (Table 2). 

## 4. Discussion

In recent years, bariatric surgical procedures have diversified and increased significantly in number. The contribution of technological developments to minimally invasive surgery is extremely important in this regard. In addition, easier information sharing has enabled surgeons to learn and start performing new laparoscopic procedures more quickly. The development of minimally invasive methods in bariatric surgery has also increased patients’ chances of treatment. Among the various bariatric procedures, Roux-en-Y gastric bypass has been performed safely for more than 40 years, and one anastomosis gastric bypass has been performed safely for more than 20 years, with long-term follow-up results [32]. Both OAGB and RYGB allow for patients to lose weight effectively by bypassing part of the stomach and small intestines. Although RYGB was the most commonly used surgical method in bariatric surgery in the past, OAGB has now become one of the frequently and more safely used methods.

Laparoscopic one anastomosis gastric bypass is also called minigastric bypass as a successful obesity treatment and was first published by Rutledge [27]. Compared to Roux-en-Y gastric bypass, OAGB began to be used as a simpler and more effective method. Despite the successful results obtained in the early period, it took a long time for it to become widespread. A significant portion of surgeons were hesitant to perform one anastomosis gastric bypass due to concerns about causing stomach and esophageal cancer [33]. However, OAGB gained popularity over time due to its short operation time and lower complication rates [32,34]. The majority of studies conducted to date have been obtained from retrospective data, and unfortunately, there are few studies conducted with randomization [30,31,35,36,37,38,39]. Long postoperative follow-up periods were not achieved in existing randomized studies. There are two studies with a follow-up period of 3 years or more comparing OAGB and RYGB, and these belong to Level et al. and Singh et al., respectively [30,31]. At the end of these two randomized studies, the number of patients remaining under follow-up was 28 and 39. To our knowledge, here we shared the third study comparing OAGB and RYGB for up to 3 years, and also in our study, 3-year follow-up was achieved in 38 patients. Randomization and long-term patient follow-up are quite challenging to achieve in bariatric surgery studies. Patients generally do not accept randomization because they are focused on a particular surgery. In addition, patients do not accompany calls to the clinic after losing weight, and long-term follow-up cannot be achieved regularly.

Katayama et al. shared 6-month follow-up results in their study; a total of 20 patients were randomized, in which they stated that approximately 70–80% of excess weight loss was achieved in 6 months [39]. Our 6-month results differ from Katayama et al. In our study, EWL% at 6 months was 62.78 ± 24.44 and 53.73 ± 16.85 in OAGB and RYGB, respectively. Level et al.’s study also included six-month EWL% results similar to ours [31]. The EWL% rates mentioned by Katayama et al. were reached only at the end of the first year in both our study and Level et al.’s study. Moreover, in Eskandaros et al.’s EWL% results, OAGB and RYGB were 41.16 ± 9.64 and 33.49 ± 8.71, respectively, at 6 months, which is incompatible with Katayama et al.’s data [39]. At the end of 12 months, they found the EWL% to be 81.67 ± 10.37 and 79.67 ± 7.25, respectively. During our 3-year follow-up period, we observed the highest EWL% rate of our patients at the end of the second year. Short follow-up does not provide realistic results in patients who have undergone bariatric surgery. It seems that follow-up of studies for at least two years may enable us to obtain more useful data.

In the YOMEGA study of Robert et al. [36], the type 2 diabetes remission rate was higher in OAGB than in RYGB, but the difference was not significant. In our study, the success rate was observed to be higher in OAGB, although there was no statistical difference. The T2DM remission rates of our patients in OAGB and RYGB were 92% and 70%, respectively. Due to the small number of patients in our study, we cannot make a strong comment about T2DM remission. Level et al. [31] conducted a randomized study with the longest follow-up period to do. The study was completed with 28 of the 33 patients included. The authors analyzed 9 OAGB and 19 RYGB patients for up to five years. In Level et al.’s study, only three T2DM patients were included, one in the OAGB group and the other two in the RYGB group. In our study, there were 13 T2DM patients in the OAGB group and 10 in the RYGB group. In the YOMEGA study, a total of 87 diabetic patients were included, and the follow-up period was 2 years. Singh et al. designed a randomized study to compare OAGB and RYGB with the remission of T2DM as the primary outcome [30]. The authors stated that the complete remission rates of T2DM were similar in both groups. At 4-year follow-up, the type 2 diabetes mellitus remission rates were 72.2% and 71.4% in OAGB and RYGB, respectively. In this regard, Singh et al.’s data and results are valuable. We found both methods to be similarly successful in other comorbidities. High blood pressure was treated in 66% of patients with both procedures. We have achieved success in the treatment of all patients with both OAGB and RYGB in asthma, sleep apnea, and dyspnea.

In the beginning, one of the most important dilemmas of OAGB was that it was a derivative of Billroth II surgery. Bile reflux and related esophageal inflammation constituted a significant part of the concerns. It has been observed that Roux-en-Y is a better choice in distal gastrectomies due to reflux [40]. The long and narrow gastric pouch in OAGB and the fact that the anastomosis is not as wide as in Billroth II increased the expectations for good results in OAGB. In addition, since the biliary limb was long, bile would be partially absorbed, and it was possible for some unabsorbed bile to be metabolized in this long intestinal segment. While de novo gastroesophageal reflux was observed in 20% of OAGB patients in our study, it was not observed in any of the RYGB patients. Similarly, Robert et al. identified bile in the stomach in 16% of patients in the OAGB group [36]. There is not yet sufficient long-term data on the long-term carcinogenic effect of OAGB-associated bile reflux. In our study, de novo reflux developed in 4 of 20 patients with OAGB. A positive clinical response was achieved with medical treatment in these patients, and there was no need for surgical intervention. On the other hand, Eskandaros et al. clarified that OAGB had favorable comparable results with RYGB in patients with obesity suffering from preoperative mild-to-moderate uncomplicated gastroesophageal reflux disease [38]. The authors stated that this may be due to a long and low-pressure gastric pouch. They also stated that the weakly alkaline pH material entering the gastric pouch can neutralize the acid that will reach the esophagus. However, they stated that alkaline reflux was significantly more reduced in RYGB than OAGB at 6 and 12 months.

One of the limitations of our study is that we did not conduct a full small bowel length measurement. The length of the biliopancreatic limb must maintain a balance between weight loss and malnutrition. We used biliopancreatic limb as 200 cm in one anastomosis gastric bypass procedure. In Roux-en-Y gastric bypass surgeries, the biliary limb was 50 cm, and the alimentary limb was 150 cm. We think that it would be more accurate to measure all bowel lengths from now on. Intestinal lengths vary from person to person, and performing an anastomosis by measuring the same limb length in each patient may cause insufficient weight loss or, on the contrary, malnutrition. According to total small bowel length measurements, no relationship between bowel length and weight could be revealed. It was found that after touching the intestines, the length of the intestine changes due to contractions. [41]. Additionally, stretched or non-stretched bowel length measurements are significantly effective in determining the length of the intestinal limbs [42]. Therefore, we believe that there is a risk in obtaining data from studies that make generalizations and make changes in biliary or alimentary limb lengths without measuring the patient’s total bowel length. We face a great difficulty in interpreting studies in which anastomoses have been made to bowel sections of different lengths without specific standardization and without specifying in detail which method was used to measure bowel length, and this situation is extremely ignored. In the method of this study, we at least stated the technique by which we measured bowel length.

Another limitation of this study is that although the physical exercises of all patients were arranged by a physical training specialist after the 2nd postoperative month, the patients did not follow a standard exercise program regularly, and this could not be recorded accurately. Physical activity also has an effect on patients’ long-term weight loss and weight maintenance on an individual basis [43,44]. 

The fact that changes in vitamin values of the patients were not compared is a limitation for the study. The main reason for this is that blood measurements could not be performed at regular intervals in the long term. In addition, another limitation of our study is the small sample size. We need further studies with larger sample sizes to obtain more robust data.

## 5. Conclusions

Both OAGB and RYGB have similar success rates in the treatment of obesity. Both surgeries are effective in treating obesity-related comorbidities. In addition to the small number of randomized studies, the number of patients in these studies is very small, and the follow-up periods are short. New studies and long-term results are needed to compare these two procedures.

## Figures and Tables

**Table 1:** Baseline characteristics of patient groups.

Operation Type	OAGB (*n* = 20)	RYGB (*n* = 18)	*p* Value
Age, mean ± SD	43.60 ± 11.10	42.72 ± 13.77	0.829 ^a^
Sex, *n* (%)			
Female	17 (85.0)	14 (77.8)	0.437 ^b^
Male	3 (15.0)	4 (22.2)	
BMI_initial, mean ± SD	44.75 ± 6.10	46.69 ± 6.62	0.353 ^a^
DM, *n* (%)	13 (65.0)	10 (55.6)	0.396 ^b^
HT, *n* (%)	9 (45.0)	6 (33.3)	0.345 ^b^
Asthma, *n* (%)	3 (15.0)	3 (16.7)	0.616 ^b^
Sleep apnea, *n* (%)	3 (15.0)	2 (11.1)	0.552 ^b^
Dispnea, *n* (%)	7 (35.0)	5 (27.8)	0.450 ^b^

^a^ Independent samples *t*-Test; ^b^ Fischer’s exact test; SD: standard deviation.

**Table 2:** Postoperative comorbidities.

Operation Type	OAGB (*n* = 20)	RYGB (*n* = 18)	*p* Value
DM, *n* (%)	1 (5.0)	3 (16.7)	0.263 ^a^
HT, *n* (%)	3 (15.0)	2 (11.1)	0.552 ^a^
Asthma, *n* (%)	-	-	NA
Sleep apnea, *n* (%)	-	-	NA
Dispnea, *n* (%)	-	-	NA
Denovo reflux, *n* (%)	4 (20.0)	-	0.066 ^a^

^a^ Fischer’s exact test.

## Data Availability

This article includes all data generated or analysed during the study.
